# Modified Fricke’s Cheek Flap for Periorbital Reconstruction: A Regional Alternative to Forehead-Based Flaps

**DOI:** 10.7759/cureus.107394

**Published:** 2026-04-20

**Authors:** Vijaykumar Huded, Gali Leela Madhuri, Parvati Huded

**Affiliations:** 1 Plastic and Reconstructive Surgery, Shri BM Patil Medical College, Hospital and Research Centre, BLDE (Deemed to Be University), Vijayapura, IND; 2 General Surgery, Shri BM Patil Medical College, Hospital and Research Centre, BLDE (Deemed to Be University), Vijayapura, IND; 3 Dentistry, PM Nadagouda Memorial Dental College and Hospital, Bagalkot, IND

**Keywords:** cheek flap, eyelid defect, modified fricke flap, periorbital reconstruction, traumatic periocular injury

## Abstract

Introduction: Periorbital reconstruction remains challenging due to the region’s complex anatomy and essential roles in eyelid function, globe protection, and facial aesthetics. Traditional reconstructive options, such as forehead-based Fricke’s flaps, may result in eyebrow distortion and poor color match. The modified Fricke’s cheek flap offers a regional alternative with improved mobility, vascularity, and aesthetic integration. This study evaluates flap survival, functional eyelid outcomes, aesthetic results, and complications of this technique in traumatic periorbital defects.

Materials and methods: A retrospective observational study was conducted at Shri B.M. Patil Medical College, Hospital and Research Centre, Vijayapura, Karnataka, India, from January 2024 to January 2025. Twenty male patients with traumatic periorbital soft-tissue loss who underwent reconstruction using the modified Fricke’s cheek flap performed by a single surgeon were included in the study. Data collected included demographics, defect characteristics, presence of fractures, flap viability, functional outcomes, cosmetic results, and postoperative complications.

Results: Road traffic accidents accounted for 18 (90%) of injuries. Superficial tissue loss occurred in all patients, while deep tissue loss was present in four (20%) patients. Associated fractures were identified in two (10%) patients. Complete flap survival was achieved in 18 (90%), with two (10%) patients developing partial distal flap necrosis, both managed conservatively. No infection, hematoma, ectropion, or wound dehiscence occurred. All patients maintained intact eyelid closure and stable contour. Donor-site morbidity was minimal, and cosmetic outcomes were clinically assessed as satisfactory in all patients, with scars concealed along natural facial creases.

Conclusion: The modified Fricke’s cheek flap appears to be a reliable and versatile option for periorbital reconstruction, demonstrating high flap survival and preserved eyelid function in this cohort. While it provides a favorable color and texture match, claims of superiority over forehead-based flaps require further comparative validation. Further comparative studies are warranted to better define its role among regional reconstructive options.

## Introduction

The periorbital region is one of the most anatomically and functionally complex areas of the face, containing structures essential for vision, eyelid closure, globe protection, tear distribution, and facial symmetry. Even small periocular defects can lead to significant functional impairment and cosmetic deformity, making reconstruction particularly challenging for surgeons [1,2].

A wide range of reconstructive options has been described for periorbital defects, including skin grafts and local or regional flaps. Forehead-based flaps are among the most commonly used options due to their reliable vascularity; however, they may be associated with eyebrow distortion, conspicuous donor-site scarring, and suboptimal color and texture match [3]. While effective, many of these procedures are technically demanding or staged.

The modified Fricke’s cheek flap represents a regional adaptation of the classical forehead-based Fricke flap, designed to provide better color and texture match, improved flap mobility, and reduced donor-site deformity [4-7]. Its proximity to the periocular region allows reconstruction with well-vascularized tissue while minimizing eyebrow distortion and visible forehead scarring [4]. Successful periorbital reconstruction requires restoration of both function and aesthetics.

In this retrospective observational study of 20 patients, we evaluate flap survival, functional eyelid outcomes, aesthetic results, and complications of the modified Fricke’s cheek flap in the reconstruction of traumatic periorbital defects.

## Materials and methods

This was a retrospective observational study, conducted at Shri B.M. Patil Medical College and Hospital, BLDE (Deemed to Be University), Vijayapura, Karnataka, India, between January 2024 and January 2025. The study was approved by the BLDE Institutional Ethics Committee (approval number: BLDE(DU)/IEC/1201/2025-26). Written informed consent was obtained from all patients, and confidentiality of patient data was maintained.

Study population

A total of 20 patients who underwent periorbital reconstruction using a modified Fricke’s cheek flap during the study period were included. Patients with extensive orbital defects requiring free flap reconstruction, prior radiation to the cheek or orbital region, uncontrolled systemic comorbidities, or active infection at the operative site were excluded. All procedures were performed by a single senior surgeon to maintain technical consistency.

Clinical data were recorded using a predefined proforma including demographic characteristics, mechanism of injury, approximate defect size (maximum dimension in cm) and location, associated fractures, depth of tissue loss, operative details, and postoperative outcomes.

Surgical technique

**Figure 1:**
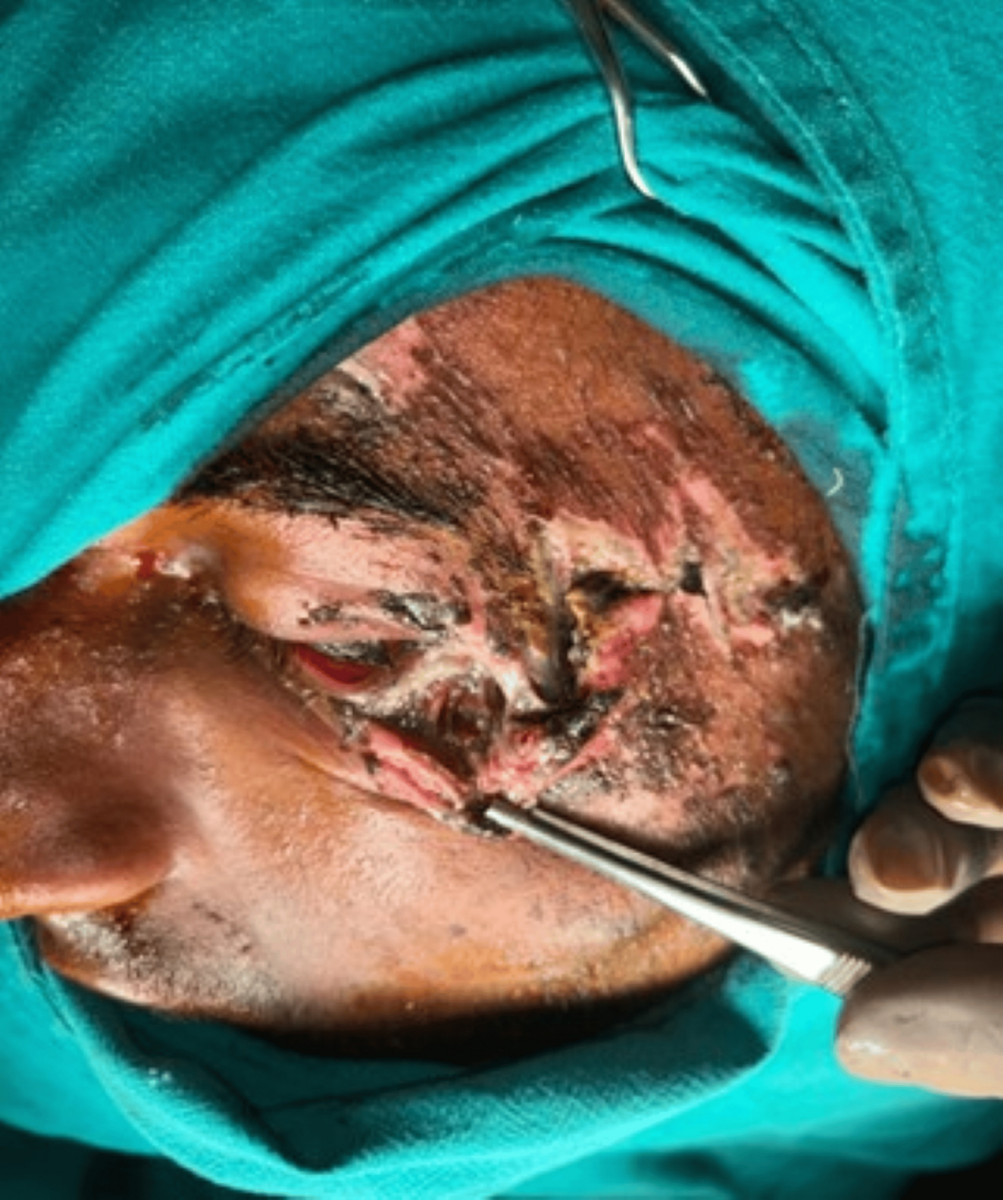
Preoperative defect following debridement Written informed consent was taken from the patient for the publication of their image in an open-access publication

**Figure 2:**
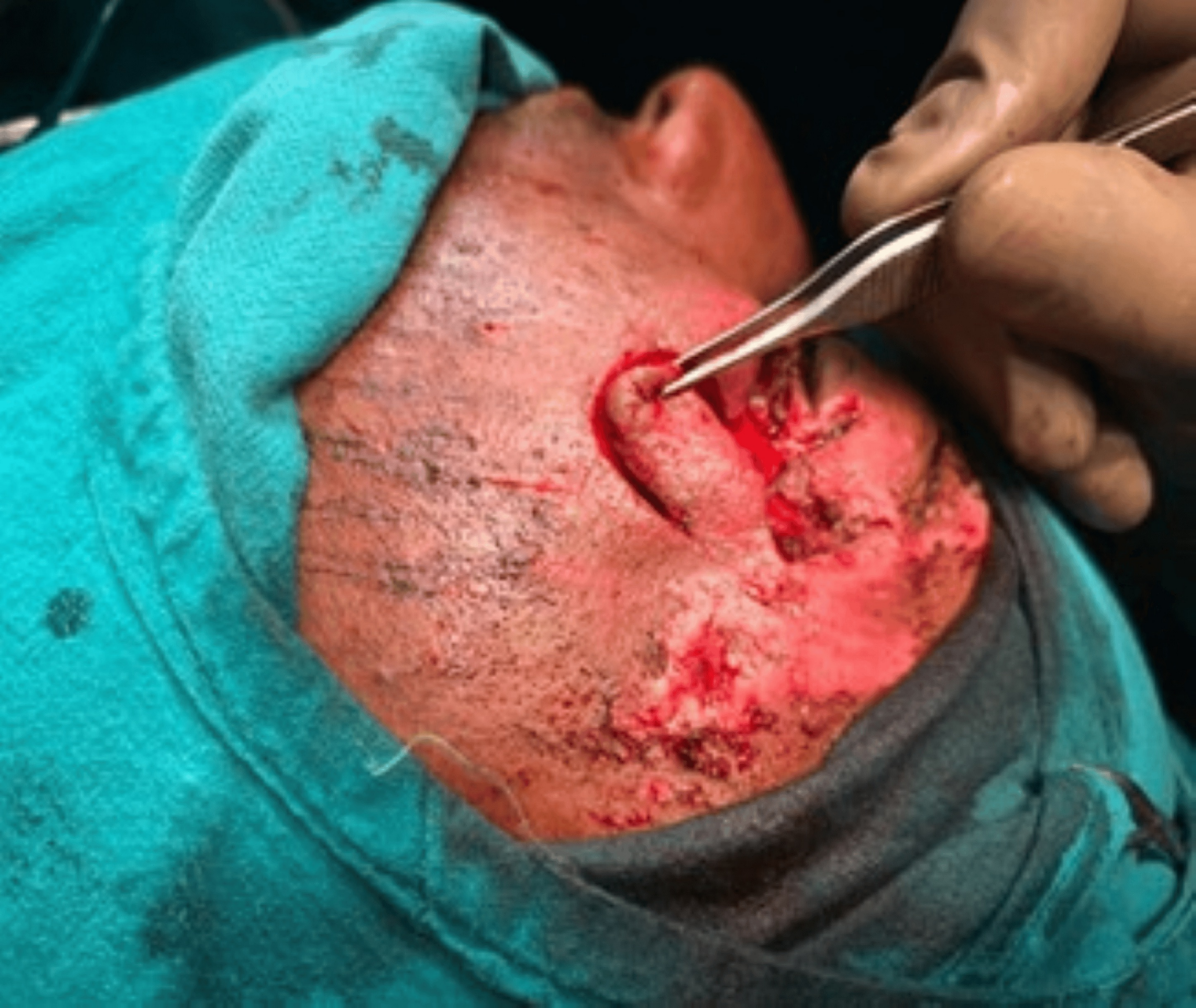
Intraoperative image post debridement and marking of flap Written informed consent was taken from the patient for the publication of their image in an open-access publication

**Figure 3:**
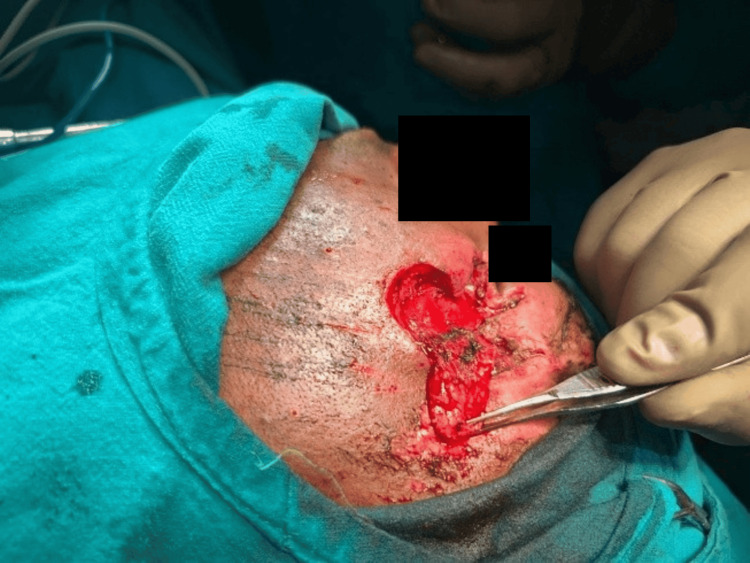
Elevation and mobilization of the modified Fricke’s cheek flap Written informed consent was taken from the patient for the publication of their image in an open-access publication

After assessment of the size, location, and orientation of the periorbital defect, flap marking was performed. An example of traumatic soft-tissue loss following debridement is shown in Figure 1. The flap length was planned to equal or slightly exceed the horizontal dimension of the defect to avoid tension during the inset. Flap width was matched to the vertical defect height to ensure adequate coverage. The flap was outlined horizontally along the infraorbital or cheek crease in accordance with relaxed skin tension lines (RSTL) to optimize scar placement (Figures 1-3).

The base of the flap (medial or lateral) was selected based on the most favorable vector of transposition and tissue availability. A broad pedicle was preserved to protect perforators. The length-to-width ratio was maintained at a maximum of 4:1, and flap width was limited by the feasibility of primary donor-site closure.

Procedures were performed under general anesthesia or local anesthesia with sedation, depending on the extent of the defect. Corneal protection was provided using lubricating ointment and a corneal shield. Key anatomical landmarks were respected: the infraorbital foramen lies approximately 7-10 mm below the orbital rim along a vertical line from the medial limbus; therefore, dissection in this region was kept superficial. The zygomatic and buccal branches of the facial nerve lie deeper, and subcutaneous plane elevation was maintained to reduce nerve injury risk.

Incision and elevation

The skin incision was made along the marked design, and the flap was elevated in the subcutaneous plane while preserving perforators and avoiding nerve injury. Wide undermining of the surrounding cheek tissue was performed to improve flap mobility and reduce inset tension. Uniform flap thickness was maintained, and the distal third of the flap was not thinned. Hemostasis was achieved using gentle bipolar cautery.

Mobilization and vector control

Retaining ligaments in the orbital and zygomatic regions, specifically the zygomaticocutaneous ligament and orbicularis retaining ligament, were selectively released as needed to allow cheek translation without inferior eyelid traction. When the defect involved or approached the lateral canthus, lateral canthopexy, canthoplasty, or periosteal anchoring sutures were placed when indicated.

Transposition and inset

**Figure 4:**
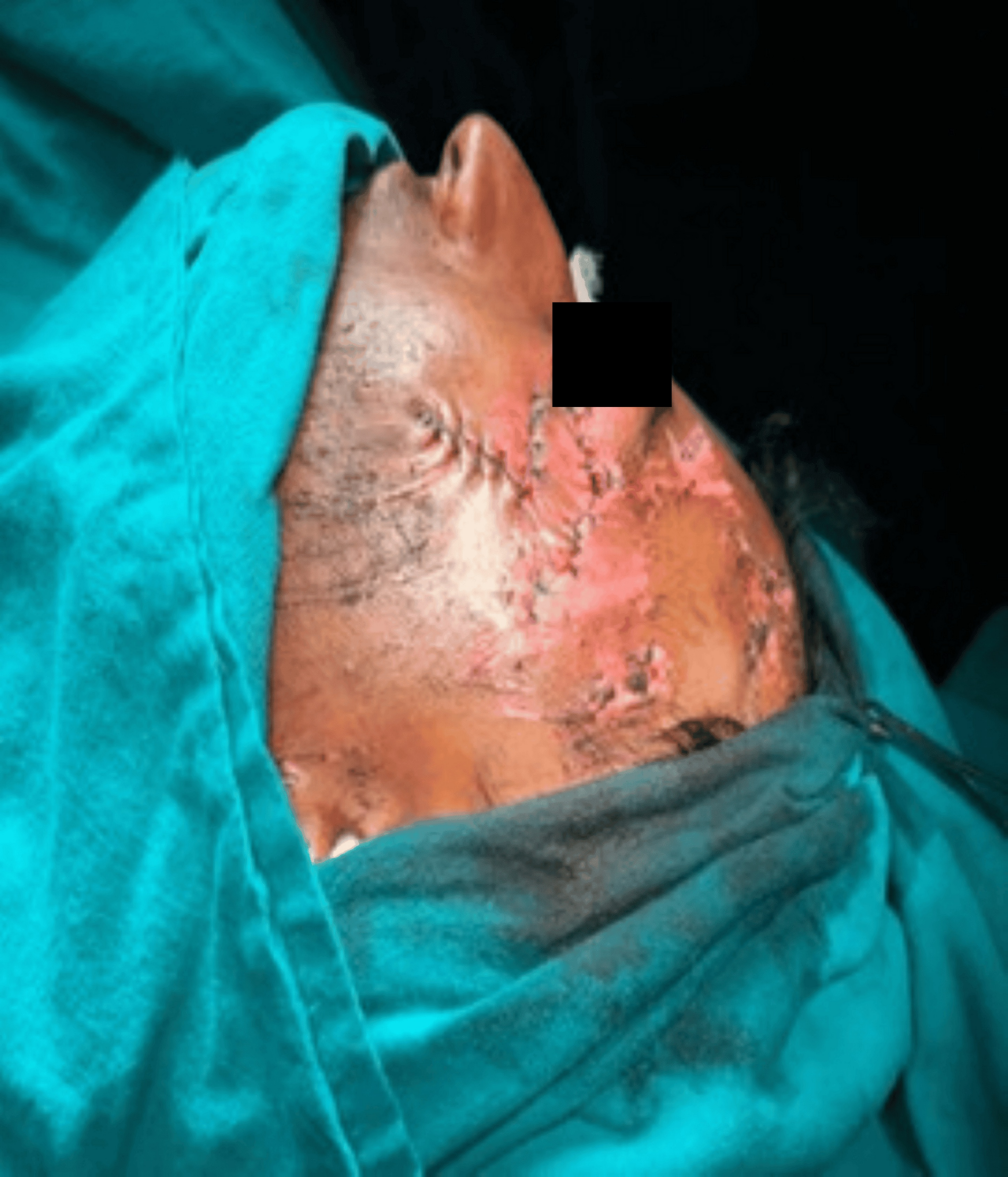
Flap transposition and final inset Written informed consent was taken from the patient for the publication of their image in an open-access publication

The flap was transposed to the defect in a tension-free curve while ensuring that the pedicle was not kinked. Final inset and donor-site closure are shown in Figure 3. Standing cutaneous cones were excised when required. For eyelid margin defects, precise anatomical alignment of the gray line, lash line, and mucocutaneous junction was performed. In cases of full-thickness defects (n=4, 20%), posterior lamellar support was provided using cartilage or mucosal grafts as indicated. Deep dermal 5-0 absorbable sutures were placed for tension distribution, and the skin was closed using fine non-absorbable sutures (6-0 for eyelid, 5-0 for cheek as required). The donor site was closed primarily (Figure 4).

Postoperative management

**Figure 5:**
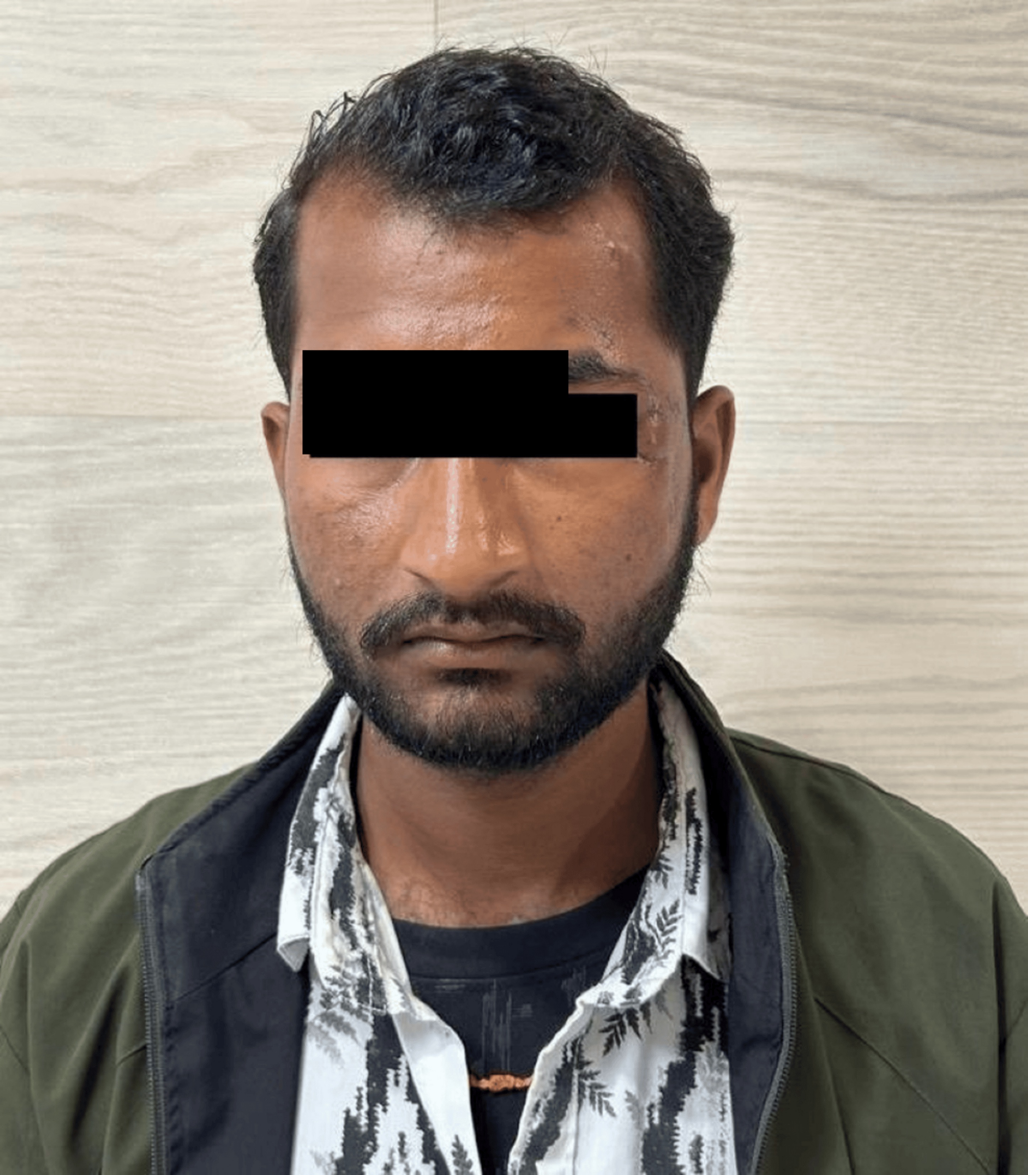
Follow-up after three months shows the wound site nearly scarless Written informed consent was taken from the patient for the publication of their image in an open-access publication

**Figure 6:**
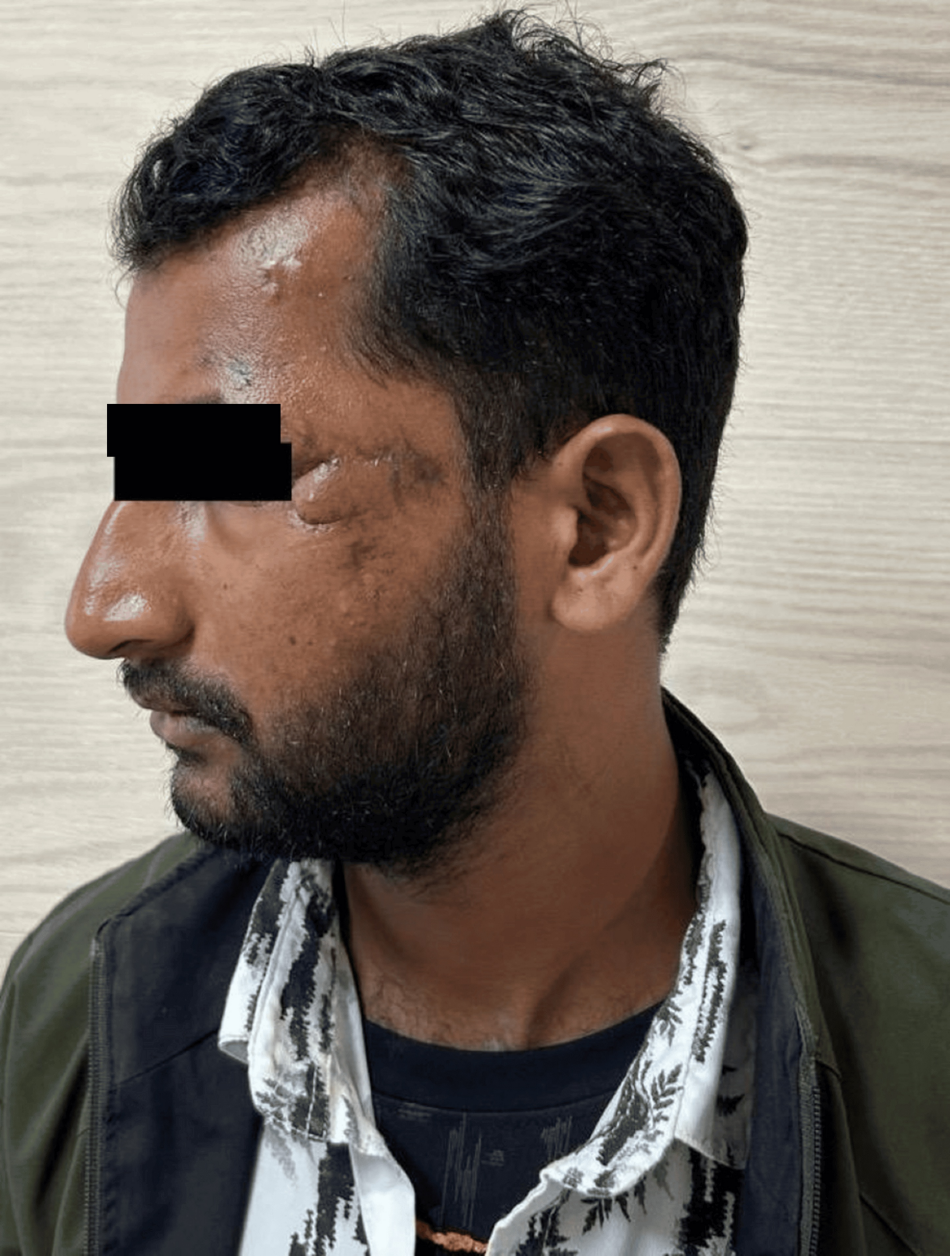
Follow-up after three months shows the wound site nearly scarless Written informed consent was taken from the patient for the publication of their image in an open-access publication

Postoperative care included wound hygiene, flap monitoring, and ocular protection. Normothermia and glycemic control were maintained as per institutional protocol. Sutures were removed between postoperative days 5 and 7. Protective eye shields were used during early healing to prevent inadvertent trauma to the reconstructed periorbital region, which is particularly vulnerable due to its exposed anatomical position (Figures 5-6).

Data analysis

Data were analyzed using IBM SPSS Statistics for Windows, Version 26.0 (IBM Corp., Armonk, New York, United States). Categorical variables were summarized as numbers and percentages, and continuous variables as mean and range. Given the observational design and sample size (n = 20), the analysis was primarily descriptive. The mean follow-up duration was three months (range: 2-5 months). Cosmetic outcomes were assessed clinically by the operating surgeon based on scar visibility, contour, and symmetry, without the use of a validated scoring system.

## Results

A total of 20 patients underwent periorbital reconstruction using the modified Fricke’s cheek flap. All patients were male (n=20, 100%). The mean age of the patients was 36 years (range: 16-56 years). Road traffic accidents were the most common etiology (n=18, 90%), followed by physical assault (2, 10%). Superficial tissue loss was present in all patients (20, 100%), while deep tissue loss was noted in 4 patients (20%). Associated facial fractures were identified in 2 patients (10%). Right-sided defects were more common (12, 60%) compared to left-sided defects (8, 40%) (Table 1).

**Table 1:** Demographic and clinical characteristics (N = 20) Data are presented as frequency (number, percentage) for categorical variables and as mean with range for continuous variables.

Parameter	Value
Sex (male), n (%)	20 (100%)
Age (years)	Mean 36; Range 16–56
Etiology, n (%)	Road traffic accident: 18 (90%)
	Assault: 2 (10%)
Soft-tissue loss, n (%)	Superficial: 20 (100%)
	Deep: 4 (20%)
Associated fractures, n (%)	Present: 2 (10%)
Laterality, n (%)	Right: 12 (60%)
	Left: 8 (40%)
Diagnosis, n (5)	Periorbital soft-tissue defect: 20 (100%)

Flap reconstruction using the modified Fricke’s cheek flap was successfully completed in all patients (n=20, 100%). Complete flap survival was observed in 18 patients (n=18, 90%), while partial distal flap necrosis occurred in two patients (n=2, 10%); both were managed conservatively. No cases of total flap loss were noted (n=0, 0%). Primary donor-site closure was achieved in all patients (n=20, 100%). Associated open reduction and internal fixation was performed in two patients (n=2, 10%).

No postoperative hematoma, wound dehiscence, infection, ectropion, or lagophthalmos was observed. Temporary chemosis was noted in a small number of patients and resolved with conservative management. Functional eyelid closure and movement were preserved in all patients (n=20, 100%). Cosmetic outcome was graded as satisfactory in all patients (n=20, 100%). No secondary revision procedures were required (n=0, 0%), and all patients completed follow-up (n=20, 100%) (Table 2).

**Table 2:** Surgical and postoperative outcomes (N = 20) ORIF: open reduction and internal fixation

Outcome Parameter	Frequency (Percentage)
Reconstruction completed	20 (100%)
Complete flap survival	18 (90%)
Partial distal necrosis	2 (10%)
Total flap loss	0 (0%)
Overall complication rate	2 (10%)
Donor-site primary closure	20 (100%)
Associated ORIF performed	2 (10%)
Functional eyelid movement preserved	20 (100%)
Eyelid malposition (ectropion/lagophthalmos)	0 (0%)
Infection	0 (0%)
Hematoma	0 (0%)
Wound dehiscence	0 (0%)
Secondary revision procedure	0 (0%)
Follow-up completion	20 (100%)
Cosmetic outcome satisfactory (clinical assessment)	20 (100%)

## Discussion

Periorbital reconstruction remains one of the most challenging areas in facial reconstructive surgery due to the complex anatomy and the critical functional and aesthetic roles of the eyelids. Achieving satisfactory outcomes requires restoring eyelid stability, maintaining globe protection, preventing exposure-related morbidity, and achieving facial symmetry. Because traumatic periocular defects are frequently associated with tissue loss, edema, and compromised vascularity, regional flaps continue to represent a practical and dependable reconstructive option [8].

In the present retrospective study, the modified Fricke’s cheek flap demonstrated high viability, with complete survival in 18 (90%) patients and partial distal necrosis in two (10%) patients, both managed conservatively. No total flap loss was observed. These findings suggest that the flap provides dependable vascularity in the setting of traumatic tissue loss. Previous studies on regional cheek and cervicofacial flaps have reported survival rates exceeding 90%, attributed to robust vascularity from branches of the facial and transverse facial arteries [9,10]. The outcomes observed in this study are consistent with these established vascular principles. Technical considerations such as preservation of a broad pedicle, subcutaneous plane elevation, limited distal thinning, and atraumatic handling were integral to flap design and likely contributed to the observed survival rates.

In cases with deep tissue loss or full-thickness eyelid defects, alternative reconstructive options include Hughes tarsoconjunctival flap, Mustardé cheek rotation flap, and free tissue transfer in extensive defects. However, these techniques are often staged, technically demanding, or associated with donor-site morbidity. In our series, the modified Fricke’s cheek flap was successfully utilized in selected deeper defects with adjunct posterior lamellar reconstruction, highlighting its applicability in carefully selected cases.

Functionally, all patients maintained preserved eyelid mobility, adequate closure, and stable contour. From a reconstructive standpoint, this is clinically significant, as insufficient lamellar support may predispose patients to complications, including ectropion, lagophthalmos, and canthal malposition [11,12]. The absence of postoperative ectropion or canthal distortion in this cohort indicates that the cheek-based flap provides sufficient anterior lamellar tissue while maintaining a favorable vector of pull, minimizing inferior traction on the lower eyelid.

Aesthetic outcomes were clinically assessed as satisfactory in all patients. The proximity of the cheek donor site to the periocular region appears to provide a favorable color and texture match when compared with traditional forehead-based Fricke flaps; however, direct comparative data are lacking. In this study, primary donor-site closure was achievable in all cases, and donor-site morbidity was limited, supporting the feasibility of this modification as a regional reconstructive option.

The flap was used predominantly in trauma-related defects, where edema and altered wound beds may complicate reconstruction. The consistent maintenance of flap viability despite these factors suggests that the modified design provides adequate vascular reliability and arc of rotation. These findings are in agreement with prior reports describing the effectiveness of cheek-based advancement and transposition flaps in periocular reconstruction, particularly in high-risk or contaminated wounds [10].

Despite these advantages, the flap has certain limitations. In extensive full-thickness defects requiring significant posterior lamellar support, the technique may need to be combined with grafts or alternative flaps. Additionally, in medially located defects or cases with limited cheek laxity, flap reach and vector may be suboptimal. The occurrence of partial distal necrosis in two (10%) cases suggests that vascular reliability, although generally high, is not absolute and may be influenced by flap design and local tissue conditions.

No late complications such as flap contracture, eyelid retraction, or contour asymmetry were observed during follow-up; however, follow-up duration was limited. Preservation of uniform flap thickness and careful protection of perforators may have contributed to stable contour and minimized contraction [13].

This study has several limitations. It was retrospective in design with a modest sample size (n = 20) and lacked a control group for comparison with other reconstructive techniques. The cohort consisted entirely of male patients, likely reflecting the demographic pattern of trauma cases, which limits generalizability of aesthetic outcomes. Cosmetic assessment was subjective and performed by the operating surgeon without validated scoring systems, introducing potential bias. Additionally, follow-up duration was relatively short, limiting assessment of long-term outcomes. The single-surgeon, single-center design may further restrict external validity. Longer follow-up and comparative prospective studies would be valuable to further define the relative advantages of the modified Fricke’s cheek flap in periorbital reconstruction.

## Conclusions

The modified Fricke’s cheek flap appears to be a reliable and versatile option for reconstruction of lateral periorbital soft-tissue defects. In this retrospective study, the flap demonstrated high survival rates, preserved eyelid function, and acceptable cosmetic outcomes in this cohort, with minimal donor-site morbidity. This cheek-based modification appears to provide favorable color and texture match while minimizing donor-site deformity.

Given its relative technical simplicity and consistent vascular reliability, the modified Fricke’s cheek flap represents a useful regional reconstructive option, particularly in trauma cases where defect geometry and tissue availability may limit alternative approaches. However, the findings should be interpreted in the context of the study’s retrospective design and modest sample size. Further comparative studies are required to validate its advantages over other reconstructive techniques.
